# Supplementary material for: Empirical methods for controlling false positives and estimating confidence in ChIP-Seq peaks
Source: BMC Bioinformatics. 2008 Dec 5;9:523. doi: 10.1186/1471-2105-9-523 (PMC2628906; doi:10.1186/1471-2105-9-523)
Supplement: Additional File 1 — A variety of html documents from the USeq web site detailing the available applications, their best usage, output file type descriptions, command line menus, etc. [file 1471-2105-9-523-S1.zip › USeqUserGuides/JavaDoc.zip!/JavaDoc/gata/aligner/package-tree.html]

gata.aligner Class Hierarchy


|  |  |  |
| --- | --- | --- |
| **Overview** \| **Package** \| Class \| Use \| **Tree** \| **Deprecated** \| **Index** \| **Help** | |  |
| **PREV**   **NEXT** | **FRAMES**    **NO FRAMES**     **All Classes** |  |


---


## Hierarchy For Package gata.aligner

**Package Hierarchies:**: All Packages

---

## Class Hierarchy

- java.lang.Object
  - gata.aligner.**AlignerPreferences** (implements java.io.Serializable)- gata.aligner.**Alignment** (implements java.lang.Comparable<T>, java.io.Serializable)- gata.aligner.**AlignParams** (implements java.io.Serializable)- gata.aligner.**Blast**- gata.aligner.**BrowseButtonAction** (implements java.awt.event.ActionListener)- java.awt.Component (implements java.awt.image.ImageObserver, java.awt.MenuContainer, java.io.Serializable)
              - java.awt.Container
                - javax.swing.JComponent (implements java.io.Serializable)
                  - javax.swing.JPanel (implements javax.accessibility.Accessible)
                    - gata.aligner.**AlignerInputPanel**
                  - java.awt.Window (implements javax.accessibility.Accessible)
                    - java.awt.Frame (implements java.awt.MenuContainer)
                      - javax.swing.JFrame (implements javax.accessibility.Accessible, javax.swing.RootPaneContainer, javax.swing.WindowConstants)
                        - gata.aligner.**AlignerInputForm**
              - gata.aligner.**DelmeAligner**- gata.aligner.**FileFocusListener** (implements java.awt.event.FocusListener)- gata.aligner.**GATAligner**- gata.aligner.**LocalAlignment** (implements java.io.Serializable)- gata.aligner.**Seqs**- java.lang.Thread (implements java.lang.Runnable)
                          - gata.aligner.**AlignerThread**

---


|  |  |  |
| --- | --- | --- |
| **Overview** \| **Package** \| Class \| Use \| **Tree** \| **Deprecated** \| **Index** \| **Help** | |  |
| **PREV**   **NEXT** | **FRAMES**    **NO FRAMES**     **All Classes** |  |


---
